# ALOPECIA AREATA AND AUTOIMMUNITY: A CLINICAL STUDY

**DOI:** 10.4103/0019-5154.41650

**Published:** 2008

**Authors:** Emy Abi Thomas, R S Kadyan

**Affiliations:** *From Department of Dermatology, Christian Medical College and Hospital, Ludhiana - 141 008, Punjab, India*

**Keywords:** *Alopecia areata*, *autoimmunity*, *concomitant diseases*

## Abstract

Alopecia areata (AA) frequently occur in association with other autoimmune diseases such as thyroid disorders, anemias and other skin disorders with autoimmune etiology. Despite numerous studies related to individual disease associations in alopecia areata, there is paucity of literature regarding comprehensive studies on concomitant cutaneous and systemic diseases. The present study has been designed to determine if there is a significant association between alopecia areata and other autoimmune diseases. This study covers 71 patients with the diagnosis of alopecia areata as the case group and 71 patients with no evidence of alopecia areata as the control group. Among the cutaneous diseases associated with AA, atopic dermatitis (AD) showed maximum frequency with an O/E ratio of 2.5, which indicates that it is two to three times more common in patients with alopecia areata. In our study, thyroid disorders showed the highest frequency with on O/E ratio of 3.2 and a *P* value of 0.01, which is statistically highly significant. Among the thyroid disorders, hypothyroidism was the most frequent association (14.1%) in our study. Since systemic involvement is not infrequent in patients with alopecia areata, it is imperative to screen these patients for associated disorders, particularly atopy, thyroid diseases, anemias and other autoimmune disorders, especially if alopecia areata is chronic, recurrent and extensive.

## Introduction

Alopecia areata (AA) is a common cause of noncicatricial alopecia that occurs as a patchy, confluent or diffuse pattern. It may occur as a single, self-limiting episode or may recur at varying intervals over many years. Strong direct and indirect evidence supports an autoimmune etiology for alopecia areata. The origin of disease process is not fully understood; however, there are indications for a T-cell-mediated autoimmune process directed against an unknown autoantigen of the hair follicle. T lymphocytes that have been shown to be oligoclonal and autoreactive are predominantly present in the peribulbous inflammatory infiltrate.^1^ Alopecia areata frequently occurs in association with other autoimmune disorders such as vitiligo, lichen planus, morphea, lichen sclerosus et atrophicus, pemphigus foliaceus, atopic dermatitis, Hashimoto's thyroiditis, hypothyroidism, endemic goiter, Addison's disease, pernicious anemia, lupus erythematosus, diabetes mellitus, Down's syndrome and others.^2^–^3^ In view of the autoimmune etiology of AA, a prospective clinical study was carried out to determine the association between alopecia areata and other autoimmune disorders.

## Materials and Methods

This study was conducted for a period of one year in the department of Dermatology of Christian Medical College, Ludhiana. During this period, a total number of 71 cases with alopecia areata were seen for associated cutaneous and systemic diseases and compared with 71 age- and sexmatched controls, who presented to our department with skin disorders other than alopecia areata such as psoriasis, lichen planus, warts, eczema, molluscum contagiosum, viral, bacterial and fungal infections.

During examination, the main site of involvement, pattern and extent of hair loss were recorded. Disease that started at or before 16 years of age was defined as juvenile disease and that at an older age as adult disease.

The diagnosis of AA was based on clinical grounds. Age at onset, duration and progression of the disease, personal and family history of atopy, family history of similar disease with special reference to autoimmune diseases and systemic complaints were noted in detail. Routine investigations such as hemoglobin, total and differential count, erythrocyte sedimentation rate and peripheral smear and special tests such as serum proteins, serum calcium, thyroid function tests and anemia panel were done in all cases. Skin biopsy, autoimmune panel, colonoscopy and stool culture were performed in selected cases. The severity of disease was defined as:^4^

1. Mild: three or less patches of alopecia with a widest diameter of 3 cm or less or the disease limited to the eyelashes and eyebrows
2. Moderate: existence of more than 3 patches of alopecia or a patch greater than 3 cm at the widest diameter without alopecia totalis or alopecia universalis.
3. Severe: alopecia totalis or alopecia universalis.
4. Snake-shaped plaques extending to the scalp border or loss of hair in the shape of a wave at the circumference of the head were defined as ophiasis.

## Results

Among the 71 patients with alopecia areata, males outnumbered females with a ratio of 2.5:1. The maximum incidence of alopecia areata was in the age group of 20-40 years (50.4%). Patients were classified according to the severity of the disease, as shown in Table 1. Thirty-eight patients had mild disease (≤3 patches or involvement of eyelashes and eyebrows), 22 patients had moderate disease (>3 patches of alopecia) and severe disease was observed in 7 patients, i.e., 4 patients of alopecia totalis and 3 patients of alopecia universalis. Ophiasic patterns of alopecia were observed in 4 patients. Eleven patients had (15.5%) a family history of alopecia areata. The family history of atopy was present in 18 patients (18.3%) and autoimmune diseases in 9 patients (12.7%). Nail involvement was observed in 12 patients (16.8%), as shown in Table 2. The commonest nail finding was pitting (7.2%), followed by longitudinal ridging (4.2%), brownish discoloration (2.8%) onycholysis (1.4%) and leuconychia (1.4%). Table 3 shows the associated cutaneous diseases in patients with alopecia areata. They include atopic dermatitis that showed maximum incidence, i.e., 14.1%, followed by neurodermatitis (7.1%), seborrhoeic dermatitis and verrucae (5.6%), vitiligo and furunculosis (2.8%) and lichen planus and lichen sclerosus at atrophicus (1.4%).

**Table 1:** Grading of alopecia areata

Type	Number	Percentage
Mild	38	53.52
Moderate	22	30.98
Severe		
A. totalis	4	5.63
A. universalis	3	4.23
Ophiasis	4	5.63

**Table 2:** Nail changes in alopecia areata

Nail changes	Number	Percentage
Pitting	5	7.2
Longitudinal ridging	3	4.2
Brownish discoloration	2	2.8
Onycholysis	1	1.4
Leukonychia	1	1.4

**Table 3:** Associated cutaneous disease

Cutaneous diseases	Study group		Control group		O/E ratio*	*P* value
				
	No.	%	No.	%		
Atopic dermatitis	10	14.1	4	5.6	2.5	0.09
Neurodermatitis	5	7.1	3	4.2	1.6	0.71
Seborrhoeic dermatitis	4	5.6	8	11.3	0.5	0.23
Verrucae	4	5.6	6	8.4	0.6	0.51
Vitiligo	2	2.8	5	7.19	0.4	0.44
Lichen planus	1	1.4	4	5.6	0.25	0.36
Lichen sclerosus et atrophicus	1	1.4	1	1.4	1	0.48
Furunculosis	2	2.8	7	9.9	0.28	0.16

* Observed by expected ratio

Of the systemic diseases observed with alopecia areata (Table 4), thyroid disorders showed the highest frequency, i.e., 18.3% with a *P* value of 0.01, which is highly significant statistically and an O/E ratio of 3.2 (O/E ratio - observed by expected ratio). Among the 13 patients who had thyroid disorders, 6 patients had severe type of AA, 3 patients had moderate type and 4 patients had mild type of the disease. Nine patients who had moderate to severe type of alopecia areata had chronic and recurrent type of the disease. Other systemic disorders noted were anemias (11.3%), diabetes (7.1%), allergic rhinitis and bronchial asthma (4.2%), hypertension and ulcerative colitis (2.8%) and SLE (1.4%).

**Table 4:** Associated systemic diseases

Systemic diseases	Study group		Control group		O/E ratio	*P* value
				
	No.	%	No.	%		
Thyroid diseases	13	18.3	4	5.6	3.2	0.01
Hypothyroidism	10	14.1	2	2.8	5	0.01
Hyperthyroidism	2	2.8	2	2.8	1	0.61
Papillary carcinoma thyroid	1	1.4	-	-	-	-
Anemias	8	11.3	3	4.2	2.6	0.12
Iron deficiency anemia	6	8.4	2	2.8	3	0.27
Pernicious anemia	2	2.8	1	1.4	2	1.0
Allergic rhinitis	3	4.2	6	8.4	0.5	0.49
Bronchial asthma	3	4.2	2	2.8	1.5	1.0
Diabetes	5	7.1	7	9.9	0.7	0.55
Hypertension	2	2.8	3	4.2	0.6	1.0
Ulcerative colitis	2	2.8	-	4.2	0.6	1.0
Systemic lupus erythematosus	1	1.4	-	1.4	1	0.48

## Discussion

Alopecia areata accounts for around 2% of new dermatological outpatient attendances in Britain and the United States. Among the many factors that appear to be implicated in etiopathogenesis are as follows: the genetic constitution of the patient, atopic state, nonspecific immune and organ-specific autoimmune reactions and possibly emotional stress.^5^

Alopecia areata has been considered as an autoimmune disease due to an aberrant T cell response against hair follicle self antigens.^6^ This autoimmune etiology has been also proposed on the basis of its association with various autoimmune diseases, the presence of auto-antibodies and various underlying immunologic abnormalities in the affected sites of these patients. Lesions in alopecia areata do not form and resolve in the usual sense; however, as evident by its chronicity and high rate of recurrence, the disease represents a continuous reactivity of the pilar units. The capacity of hair to react in clinically recognized patterns as alopecia areata is probably inherited although the mode of inheritance, penetrance and expressivity of the genes must be highly variable.^3^ Alopecia areata may begin as early as the first month of life^7^ or as late as in the late seventies.^3^ The age of the youngest patient in our study group was 8 years and that of the oldest was 58 years. Evidence regarding a genetic etiology comes in the form of a familial incidence of AA that varies from 10 to 20% in different studies.^3^,^8^ HLA studies have shown that HLA-AI, HLA-DQ1 and HLA-DQ3 were significantly frequent in patients with alopecia areata than in controls. They also found that HLA-DR 16 was significantly less common in patients with alopecia areata than in the control group and concluded that this allele might have a protective role for alopecia areata.^4^ In HLA-alopecia areata association, ethnic differences may play a role.^4^ Family history of alopecia areata was present in 15.5% of our patients, which is in agreement with the study done by Muller and Winkelmann,^3^ where they found 18% cases with family history of alopecia areata. Similarly, the family history of alopecia areata was positive in 10.4% of patients studied by Sharma *et al.*,^9^ however, in another Indian study, the family history of alopecia areata was low, i.e., 1.5%.^5^

Atopy has been reported to occur with an increased frequency in patients with alopecia areata.^3^,^10^ The atopic type in Ikeda's classification of alopecia areata comprised 10% of 1989 patients.^11^ Similarly 11% of 736 patients of alopecia areata studied by Muller and Winkelmann had concomitant asthma or atopic dermatitis.^3^ In the present study, atopy was associated with increased frequency in patients with alopeciaareata, i.e., 22.5% cases, which is in agreement with the frequency of atopic manifestations in patients with alopecia areata ranging from 1-57% in a study performed by Penders.^12^ Atopic disorders have been reported to occur with an incidence of 1-52% in patients with AA.^13^–^16^ Among the cutaneous diseases associated with AA, atopic dermatitis showed maximum frequency with an O/E ratio of 2.5, which indicates that it is two to three times more common in patients with alopecia areata.

Atopic dermatitis is a classic endogenous eczema, which is a pruritic, recurrent, flexural and symmetric eczematous dermatosis. The clinical manifestations depend on the age of the patient. Infants present with facial and body lesions, either focal or generalized. In adolescents and adults flexural areas and the hands are mainly involved.^17^ Muller and Winkelmann^3^ and Ikeda^11^ have stated that patients of alopecia areata with atopy have earlier age of onset with severe form and longer duration of disease, but compared to these observations, we observed no relationship of associated atopic disorders with age of onset and severity of disease as 12 patients who had associated atopy had mild to moderate type of AA as compared to 4 patients who exhibited ophiasic patterns. In a study from Chandigarh, no relationship was observed between presence of atopy and an early onset of an increased severity of alopecia areata.^14^ In a study by Kaur and Sharma *et al.*,^18^ despite the increasing frequency of severe alopecia in atopics, the results did not attain statistical significance. In their study, 50% of patients had evidence of atopy, allergic rhinitis being the commonest in 42 patients or first degree relatives followed by bronchial asthma in 6 and atopic dermatitis in 2, and they concluded that associated atopy apparently does not affect the severity of alopecia areata in North Indians. It is possible that severity of associated atopic disorders may be a critical factor in determining the severity of AA than the mere presence of an atopic disorder.^18^

One of the main systemic associations of autoimmune disease is with thyroid abnormalities. The incidence of thyroid disease has varied from 8 to 28% in patients with AA.^14^ Lewinski and Broniarczyk-Dyla *et al.* also confirmed the frequent coexistence of alopecia areata and thyroid abnormalities.^19^ Conversely, in 1994, Puavilai *et al.* estimated that the prevalence of thyroid disease is relatively low (7.2%) and they were not statistically different from patients with AA and control group.^20^ In our study, thyroid disorders showed the highest frequency with an O/E ratio of 3.2 and a *P* value of 0.01, which is highly significant statistically and correlates with the previous reports. Among the thyroid disorders, hypothyroidism was the most frequent association in our study (14.1%). Although the effects of hypothyroidism on hair have long been known, the mechanism has not been elucidated.^21^ Changes in the texture of the hair as well as alopecia of scalp, eye brow and other body hair are recognized as characteristic clinical signs of myxedema. Reversible alopecia has also been noted in iatrogenic hypothyroidism that occurs during the treatment of thyrotoxicosis (Grave's disease). In several reports, this type of hair loss has been attributed to toxic effects of antithyroid drugs such as thiourylenes.^22^–^24^ Failure or delay in the resumption of anagen undoubtedly occurs in hypothyroidism since there is loss of hair without replacement. Such an effect could contribute an increase in telogen hair counts as increasing numbers of club hairs accumulate in dormant follicles before they are gradually shed. The regrowth of hairs after replacement therapy with thyroid hormone reiterates the reversibility of this phenomenon. Not all patients with hypothyroidism have alopecia; thus, it is likely that the magnitude of the effect of thyroid hormone on hair growth is variable and its expression may be conditioned by local factors and other hormonal influences.^25^

Other associated disorders in our study were vitiligo, lupus erythematosus and ulcerative colitis, which has also been reported by many authors.^2^,^26^ The high frequency of anemias in our study is in agreement with the study performed by Kern *et al.*, where he found a statistically significant association between alopecia areata and pernicious anaemia.^27^ Although our study did not show statistically significant association with anemia, it may be clinically significant and probably due to the smaller study group and has to be corroborated by studying a larger group. The disorders of autoimmune pathogenesis occur with increased frequency in patients with a history of another autoimmune disease. The tendency to develop another disease occurs in approximately 25% of these patients.^28^ Multiple autoimmune syndromes can be classified into 3 groups according to the prevalence of their associations with one another.^29^ Type 1 comprised myasthenia gravis, thymoma, polymyositis and giant cell myocarditis. Type 2 includes Sjögren's syndrome, rheumatoid arthritis, primary biliary cirrhosis, scleroderma and autoimmune thyroid disease. Type 3 groups together autoimmune thyroid disease, myasthenia and/ or thymoma, Sjogren's syndrome, pernicious anemia, idiopathic thrombocytopenic purpura, Addison's disease, insulin dependent diabetes, vitiligo, autoimmune hemolytic anemia, systemic lupus erythematosus and dermatitis herpetiformis.

In the final analysis, we agree with the opinion of Muller and Winkelmann^3^ that autoimmune disorders are more commonly associated with alopecia areata and that the basis of such association is the formation of organ-specific autoantibodies that may play a pathogenic role in both the disorders. Our study group showed significant association with thyroid disorders and a higher percentage of these patients, i.e., 9 out of 13 patients, who had associated thyroid disorders had moderate to severe type of alopecia areata that was chronic and recurrent. These observations highlight the significance of screening for thyroid abnormalities in patients with chronic, recurrent and extensive type of alopecia areata.
